# RNA editing of the 5-HT2C receptor in the central nucleus of the amygdala is involved in resilience behavior

**DOI:** 10.1038/s41398-021-01252-7

**Published:** 2021-02-24

**Authors:** Gal Warhaftig, Chaya Mushka Sokolik, Khen Khermesh, Yehuda Lichtenstein, Michal Barak, Tzofnat Bareli, Erez Y. Levanon, Gal Yadid

**Affiliations:** 1 The Mina & Everard Goodman Faculty of Life Sciences, Bar-Ilan University, Ramat-Gan, 52900 Israel; 2 Leslie Susan Gonda (Goldschmied) Multidisciplinary Brain Research Center, Bar-Ilan University, Ramat-Gan, 52900 Israel

**Keywords:** Molecular neuroscience, Learning and memory

## Abstract

Post-traumatic-stress-disorder (PTSD) is a stress-related condition that may develop after exposure to a severe trauma-event. One of the core brain areas that is considered to be a key regulatory region of PTSD is the amygdala. Specifically, the central amygdala (CeA) is involved in emotion processing and associative fear learning memory, two main circuits involved in PTSD. Long term dysregulation of trauma-related emotional processing may be caused by neuroadaptations that affect gene expression. The adenosine-(A) to-inosine (I) RNA editing machinery is a post-transcriptional process that converts a genomic encoded A to I and is critical for normal brain function and development. Such editing has the potential to increase the transcriptome diversity, and disruption of this process has been linked to various central nervous system disorders. Here, we employed a unique animal model to examine the possibility that the RNA editing machinery is involved in PTSD. Detection of RNA editing specifically in the CeA revealed changes in the editing pattern of the 5-HT2C serotonin receptor (5-HT2CR) transcript accompanied by dynamic changes in the expression levels of the ADAR family enzymes (*ADAR* and *ADARb1*). Deamination by *ADAR* and *ADARb1* enzymes induces conformational changes in the 5-HT2CR that decrease the G-protein-coupling activity, agonist affinity, and thus serotonin signaling. Significantly, a single intra-CeA administration of a 5-HT2CR pharmacological antagonist produced a robust alleviation of PTSD-like behaviors (that was maintained for three weeks) as well as single systemic treatment. This work may suggest the way to a new avenue in the understanding of PTSD regulation.

## Introduction

Post-traumatic stress disorder (PTSD) is a trauma- and stress-related disorder that may develop in survivors of a life-threatening traumatic event and can cause intense fear and a feeling of helplessness^1^. Currently, PTSD is defined by the coexistence of four symptoms that may be evoked initially by the traumatic event itself^2^, but then may increase over time, in response to stress-associated cues, despite the absence of further exposure to stress^3^. Although the exact neuronal mechanism that underlies PTSD is yet to be discovered, a wealth of data concerning the biological circuits involved in fear and anxiety implicate the amygdala as a region that is central to these behaviors^4^. The amygdala has been shown to participate in the acquisition of conditioned fear paradigms in animal studies^5,6^ and in combat veterans, if this region is damaged, the development of PTSD is neutralized^5^.

Whereas the amygdala has a number of nuclei with diverse activities, the central nucleus of the amygdala (CeA) deserves special attention due to its role in mediating the response to negative states associated with stress^7,8^. A number of animal studies involving fear learning reported that damage to the CeA causes a deficit in conditioned orientating and disrupts the fearful associative learning process^9^. These results have implicated the CeA as an important mediator in the physiological and behavioral expression of conditioned fear^5^. In particular, the serotonergic system that projects to the amygdala has been linked to PTSD and emotional regulation^10^ and has been implicated in the pathophysiology of mood and anxiety disorders^11,12^.

Moreover, the CeA is known to be involved in an innate- and learned-fear as well as in the regulation of freezing behavior-responses^13,14^. Although neither a single gene nor a single signaling pathway region may entirely account for the development of a complex disease, low serotonergic activity and brain regional abnormalities in serotonin neurotransmission have been proposed as biological traits related to depression and suicidal behavior^15^. Among the serotonin receptor family, the serotonin 2c receptor (5-HT2CR) is of interest. Recent studies examining the effect of 5-HTR2C agonists showed that the CeA is highly sensitive to their effect on dopaminergic release, emphasizing the importance of this receptor located in the CeA in different neuropsychiatric diseases^16,17^. On the other hand, 5-HTR2C was suggested to play an important role in preventing repeated restraint stress in the amygdala^18^ and has been associated with regulation of mood, appetite, sleep, and sexual behavior^19,20^. Altered activity of the 5-HTR2C, a G-coupled protein receptor, has been reported in a variety of neuropsychiatric disorders^21^. For example, viral-overexpression of this receptor in the amygdala resulted in an anxiogenic effect^22^, which could be counteracted, at least in the short term, by the injection of a pharmacological 5-HT2CR antagonist directly into the amygdala^23^.

However, the long term effects of exposure to a severe traumatic event, even in the absence of further stress, have not been much studied^3^. Interestingly, the 5-HT2CR is a target of post-transcriptional adenosine-to-inosine (A-to-I) RNA editing^24,25^ carried-out by the adenosine deaminases acting on RNA enzymes (*ADARs*) family. The editing modifies an A to an I, leading to alterations in the amino acid sequence, and can generate a diversity of proteins that are different from those encoded in the genomic-DNA. It was previously shown to alter the 5-HT2CR affinity to its ligand through this machinery^25–29^. Nonetheless, the cellular and molecular targets in the CeA underlying the specific effect of RNA editing of the 5-HTR2C are still inconclusive^30^.

Since PTSD is characterized as a memory disorder^31^, and the neuroadaptation dynamic of learning and memory may be responsible for the distressing memories of an emotionally traumatic event, we hypothesize that the regulation of gene expression in the CeA may contribute to the enduring plasticity of PTSD^32^. If 5-HT2CR- RNA editing in the CeA indeed plays a causal role in PTSD-like behavior, then targeting the dynamic changes in this pathway could be expected to influence the PTSD-like behavioral phenotype. A variety of experimental protocols have been designed to follow long-lasting responses to fear in rodents over periods of 24 h to 7 days post a single traumatic exposure^33–39^. Nonetheless, the vast majority of these protocol studies do not monitor the behavioral manifestation longer than the initial traumatic event^40,41^.

In the current study, we used an established animal model for the study of PTSD, which mimics the clinical expression of PTSD, including anxiety, social avoidance, and hyperarousal behavior^40–43^, and in addition, provides the opportunity to examine the effects of the action of the RNA editing machinery on the 5-HT2CR longitudinally to exposure to a traumatic event. Specifically, we aim to test whether the CeA-*ADAR* enzymes are involved in PTSD-like susceptibility and resilience behaviors and to track any consequent changes in the A-to-I RNA editing patterns of the 5-HT2CR. Detection of such changes may reveal a role for the RNA editing machinery in PTSD-like susceptibility to a trauma-related memory.

## Methods

### Animals

Adult male Sprague–Dawley rats (250–270 g; Envigo, Rehovot, Israel) were housed under conditions of constant temperature (22 °C) and 50% humidity, with a 12-h light–12-h dark cycle. The rats were allowed to habituate to the animal house for one week before beginning the experiments. Rats were housed three per cage, where two of the animals were experimental rats, and the third was a companion rat. The same three rats remained together until the end of the study. Food and water were provided *ad libitum*. All experiments were performed between 07:00 and 17:00, in daylight. All animal procedures were approved by the Bar-Ilan University Animal Care Committee and were carried out in accordance with the NIH Guide for the Care and Use of Laboratory Animals.

### Behavioral measurements

**Fig. 1:**
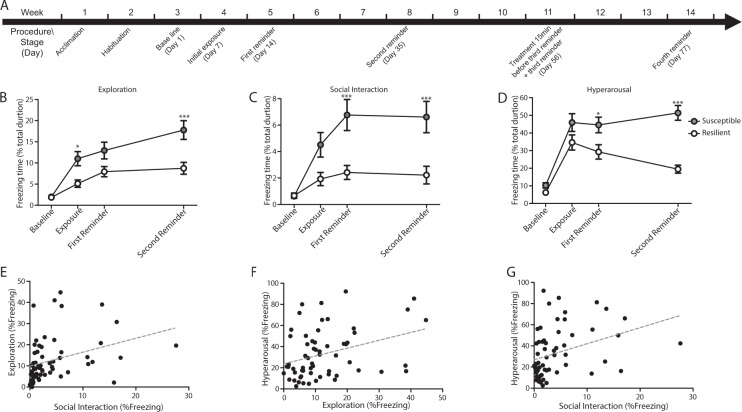
Behavioral response in the PTSD model. (**A**) Schematic depiction of the experimental model. The animals began the experimental procedure after two weeks of acclimation and habituation to the home cage and the open field. Baseline response to the three behavioral scenarios was monitored on day 1. Day 7: Exposure to ‘trauma’ (Initial exposure). Day 14: first exposure to the ‘trauma’s’ reminder (‘first reminder’). Day 35: second exposure to the ‘trauma’s reminder (‘second reminder’). Day 56: third exposure to the ‘trauma’s reminder (‘third reminder’). Day 77: fourth exposure to the ‘trauma’s reminder (‘fourth reminder’). (**B**) Freezing behavior during: exploration, (**C**) social interaction, and (**D**) hyperarousal tests. After exposure to trauma and the reminders, susceptible but not resilient animals showed an increase from baseline in freezing behavior, i.e., incubation of fear over time in all three behavioral tests (exploration: *p < 0.05 and ***p < 0.001 susceptible vs. resilient; social interaction: ***p < 0.001 susceptible vs. resilient and hyperarousal: *p < 0.05 and ***p < 0.001 susceptible vs resilient). Data presented as mean ± SEM; *n* = 26–35 per group. (**E**–**G**) Pearson’s product-moment correlation between the following tests: (**E**) exploration×social interaction, (**F**) hyperarousal×exploration, and (**G**) hyperarousal×social interaction (*r* > 0.33; *p < 0.005).

The PTSD-animal model, based on Kesner et al. and Elharrarr et al.^41,42^, consists of several stages spread over 8 weeks (Fig. 1A and Supplementary Materials and Methods). Briefly, adult male Sprague–Dawley rats were exposed to a predator-associated ‘trauma’ (cat scent) and then placed in an open field. The freezing response was monitored, during a series of three clinically relevant behavioral scenarios (5 min each): (a) situated alone (‘exploration’), (b) with a habituated companion animal (‘social interaction’), and (c) during post-startle response after exposure to loud noise (‘hyperarousal’). Baseline response to the three behavioral scenarios was monitored on day 1 (baseline). Subsequent behavioral responses were measured 7 days after the initial exposure to the trauma (bedding with cat scent), and then after reminders of the trauma (litter with the same texture but without predator bedding), on day 14 (first reminder) and day 35 (second reminder).

### Behavioral data analysis

Baseline behavioral data were analyzed by Explore (SPSS 11), to define the range for each basal behavioral parameter within the population. The upper and lower levels of this range were considered the boundaries of the ‘normal’ baseline. Deviations from this range were used to define PTSD-like behavior retrospectively (after the conclusion of the second reminder testing). According to the results, animals were categorized either as ‘susceptible’ (exhibiting PTSD-like behavior above baseline under all three conditions) or ‘resilient’ (exhibiting at least one behavior within baseline range)^41,42^.

### RNA and cDNA preparation

Brains were removed immediately after the second reminder and were placed in a perspex brain matrix and sliced into 1.0 mm segments. The CeA was punched using a 13G-14G microdissecting needle and was frozen at −70 °C until the RNA was extracted using the Total RNA Purification Micro Kit (Norgen biotek CORP, Canada) according to the manufacturer’s instructions. Extracted RNA (2 µg) was treated with DNase and reverse-transcribed to generate cDNA (qScript cDNA Synthesis, Quanta BioSciences).

### Amplification of the target regions containing the target editing sites using the Fluidigm Access Array Microfluidic system

To precisely detect and measure the levels of A-to-I RNA editing in susceptible and resilient animals, targeted amplicons were generated and barcoded using a two-step PCR strategy, which also minimized the total number of primers required. The specific primers of the targeted genes were designed using Primer 3.0 (http://frodo.wi.mit.edu) and were tested for specificity and sensitivity by PCR before they were included in the primers set. Quantification of multiple RNA editing sites was followed by next-generation sequencing (see Supplementary Materials and Methods for full details).

### Fluidigm library sequencing

Libraries were pooled and sequenced on Ion-Torrent PGM using the Ion PGM Sequencing 200 Kit v2 and the 1G-Ion 318 Chip Kit v2, all according to the manufacturer’s instructions (Life Technologies). The sequencing adaptors and tag barcodes that were attached to each PCR product (amplicon) were used to identify each sample by the sequence in the Fluidigm access-array library, prepared as described in the Supplementary Materials and Methods.

### Bioinformatic sequence analysis and cluster analysis of the 5-HT2CR isoforms

We used the UCSC genome browser Rat. 2004 (Baylor 3.4/rn4) assembly to identify any discrepancies between the RefSeq RNA data and the RNA sequencing output. For our focused screen, we employed a targeted-sequencing variation of next-generation sequencing (NGS) to generate and sequence multiple PCR amplicons from pre-determined genes, which contained the target editing site/s. The data obtained were screened for any A/G mismatches within the cDNA sequences. The signal strength of such mismatches was summed and scored according to the overall coverage as manifested by the output number of reads, and more importantly, by the percentage of A-to-G levels (see Supplementary Materials and Methods for full details). For cluster analysis of the 5-HT2CR, we detected the editing sites of the 5-HT2CR in each read. Next, we joined all the isoform combinations of the editing sites of the receptor and calculated the abundancy of each isoform from the total number of reads (see Supplementary Materials and Methods for full details).

### qRT-PCR Analysis

Expression levels of 5-HT2CR, *ADAR* and *ADARb1* were assessed by qRT-PCR of total RNA extracted from the CeA and reverse transcribed to generate cDNA. The qRT-PCR reactions were carried out on a Step One Plus Real-time PCR system using fluorescent SYBR Green fast mix technology (see Supplementary Materials and Methods for full details).

### Western blot protein analysis

Whole-cell proteins were extracted from the central amygdala and analyzed by western blot to evaluate the protein levels of *ADAR* and *ADARb1* (see Supplementary Materials and Methods for full details).

### Central amygdala intracerebral injection of RS-102221

After the second reminder (day 35), susceptible and resilient animals were anesthetized by intraperitoneal administration of ketamine hydrochloride (100 kg/mg) and xylazine (10 mg/kg). Each experimental group was randomly divided into two sub-groups, then the animals in each group were implanted bilaterally with a guide cannula (30 gauge) placed 1 mm above the CeA, sealed with a cannula dummy (Plastics One), and secured to the skull with screws and dental acrylic cement. The coordinates of the cannula, relative to Bregma^44^, were: CeA: anterior –2.56, lateral –4/+4, ventral –7 mm. Correct placement was achieved by using a computer-guided stereotaxic instrument and a motorized nano-injector (Angle Two Stereotaxic Instrument, St. Louis, Missouri). The selective 5-HT2CR antagonist RS-102221 (Tocris, Bristol, UK) was injected (total of 0.2 ul, 0.02 ul/min per side) through the cannula 15 min before the third reminder (day 11, see Supplementary Materials and Methods for full details).

### Fluorescent staining

Cannula placement was verified by histological examinations of brain sections stained with propidium iodide (PI) (see Supplementary Methods for full details).

### Statistical analysis

Data were analyzed by two-way ANOVA, one-way ANOVA, and Student’s-*t* test (see Supplementary Methods for full details).

## Results

### Distinct freezing behavior characterization of ‘susceptible’ and ‘resilient’ animals exposed to trauma

Freezing behavior of Sprague-Dawley rats (*n* = 60) exposed to ‘trauma’ and three subsequent trauma-associated reminders (Fig. 1A) was measured under three behavioral scenarios—exploration, social interaction (with in-house companion rat), and hyperarousal. The PTSD-like behavior of each animal was then compared with the baseline and with the range of the population in the three behavioral tests (Fig. 1B–D, for full details, see Supplementary Materials and Methods, behavioral procedure and statistical analysis sections). The results allowed us to unambiguously distinguish two subpopulations, namely, resilient (*n* = 35) and susceptible (*n* = 25) animals^41,42^. After experiencing the traumatic event and the related reminders, susceptible animals showed an increase in freezing behavior over time in all three behavioral tests, while the resilient group did not. Analysis of all baseline behavioral samples revealed that the upper level for excluding outliers (95% confidence) in the exploration, social interaction, and hyperarousal conditions was twice the interquartile range. Animals in the susceptible and resilient groups showed a high within-group correlation (Fig. 1E–G, for full details, see Supplementary Materials and Methods, statistical analysis sections).

### Distinct A-to-I editing of the 5-HT2CR in the CeA of susceptible and resilient animals

The CeA of animals retrospectively categorized as susceptible or resilient was punched immediately after the second trauma reminder (day35), and samples were subjected to the A-to-I RNA editing detection assay. Based on the results of Pinto and colleagues^45^, we assayed 48 RNA editing sites selected primarily because of their mammalian conservation^45^. In order to improve the accuracy of quantification of RNA editing levels (%), we discarded all the measurements with the cutoff of read coverage < 700 and A-to-I editing levels < 5%. Hence, only editing sites above this threshold and with a detectable signal in at least 5 animals in each group were selected for subsequent statistical analysis. The ten sites out of 48 that passed these criteria are located in the 5-HT2CR (ChrX: 118431948, 118431950, 118431955, and 118431960), Glutamate Ionotropic Receptor Kainate Type Subunit 2 (Grik2- Chr20: 55549608, 55549612, and 55579573), Calcium Voltage-Gated Channel Subunit Alpha1 D (CACNA1D- Chr16: 6068246 and 6068254), and Component of Oligomeric Golgi Complex 3 (Cog3- Chr15: 61477446).

**Fig. 2:**
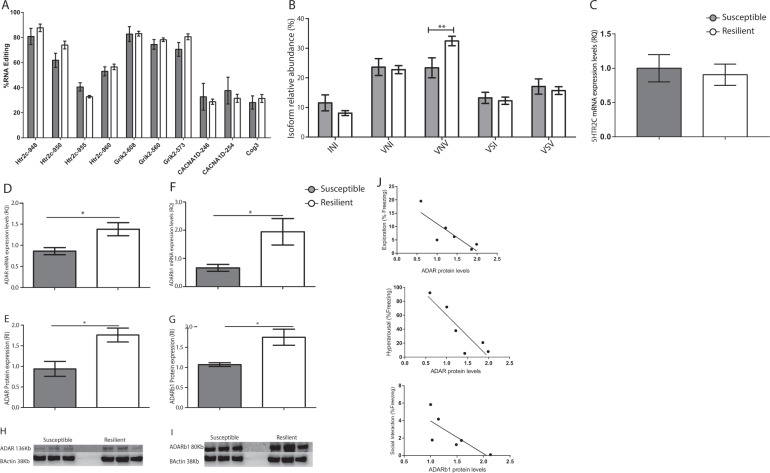
Targeted sequencing by mmPCR revealed differential RNA editing of the 5-HT2CR in the CeA of susceptible compared to the resilient group. (**A**) The ten RNA editing sites common to both experimental groups that passed the defined criteria. No significant differences were found between the experimental groups (*p* > 0.05) in RNA editing levels in the sites of the 5-HT2CR, Grik2, CACNA1D, and Cog3. (**B**) Relative abundance of the 5-HT2CR mRNA variants produced by RNA editing. Data show a relatively higher abundance of the VNV isoform (*p < 0.05) in the resilient animals than in the susceptible group. Data presented as mean + SEM; *n* = 7–13 per group. (**C**) mRNA expression levels of the 5-HT2CR did not reveal a significant change between the experimental groups (*p* > 0.05). (**D**, **F**) qPCR analysis revealed significantly higher expression levels of *ADAR* (**D**) and *ADARb1* (**F**) in the resilient group compared to the susceptible group (*p < 0.05). Data presented as mean + SEM; *n* = 6 7 per group. (**E**, **G**–**I**) Protein expression of *ADAR* (**E**, **H**) and *ADARb1* (**F**, **I**) analyzed by western blot with specific antibodies against *ADAR*, *ADARb*1 and β-Actin as a loading control revealed a significantly elevated expression of these proteins in the resilient animals compared to the susceptible group (*p < 0.05). Data presented as mean + SEM; *n* = 3 per group. (**J**) Pearson’s correlation between ADAR proteins and freezing behavior at the second reminder time point presenting a high correlation between: *ADAR* and exploration (*r* = −0.7, *p* = 0.04), *ADAR* and hyperarousal (*r* = −0.7 and *p* = 0.02), and *ADARb1* and social interaction (*r* = −0.6 and *p* = 0.07).

Statistical analysis did not reveal any significant differences in the CeA-edited sites of susceptible compared to resilient animals (*p* > 0.05, Fig. 2A). Interestingly, 4 out of the 10 edited sites were found to be located within the 5-HT2CR which are known to have close genomic proximity. This receptor has five different editing sites: A-D and C’ (which is the rarest site in rats and humans) that span over 14 nucleotides on chr X^46,47^. The sites are located in the second intracellular loop of the G-protein coupled receptor and may, therefore, modulate the serotonin neurotransmission signaling cascade^26,48^. The edited isoforms were shown to have a robust reduction of the agonist-stimulated G-coupled protein compared to the non-edited form of the receptor^48,49^. Moreover, RNA editing also led to a loss of the active state of this receptor^25^ and a delay in agonist-stimulated calcium release in the fully edited isoforms^27^. The ability to regulate the RNA-editing of these five sites can, therefore, be expected to generate high diversity by generating up to 32 different mRNA transcripts that may then encode as many as 24 different protein isoforms that vary in their biochemical properties.

Our evaluation of the RNA editing levels of the four major 5-HT2CR sites (A-D) in the CeA did not uncover any significant site-specific differences between the susceptible and resilient animals (Fig. 2A). However, analysis of the isoform frequency (%) generated by the different combinations of the editing process regulated by *ADAR* and *ADARb1* revealed a significant increase (*p < 0.05) in the partially edited VNV isoform in the resilient group compared to the susceptible group (Fig. 2B, see Supplementary Methods and Statistical analysis for full details). This isoform has 2-3 sites that are being edited simultaneously (sites A + D and A + B + D)^50^. In order to evaluate whether the VNV isoform abundance was not due to differences in the mRNA expression levels of the receptor, we measured its expression levels in susceptible and resilient groups. This analysis did not reveal any significant changes in the expression levels of the 5-HT2CR of the experimental groups (*p* > 0.05) (Fig. 2C).

### Relative expression of ADAR and ADARb1 in the CeA of susceptible and resilient animals

We next tested whether the observed higher frequency of the VNV isoform was associated with increased expression of the enzymes of the *ADAR* family that regulate the RNA editing process (Fig. 2D–I): *ADARb1*, which acts on sites A and B of the 5-HT2CR^51^, and *ADAR*, which edits site D of the 5-HT2CR^52^. qPCR analysis revealed a significant increase in the mRNA expression levels of *ADAR* and *ADARb1* in the resilient group compared to the susceptible group (*p < 0.05, Fig. 2D and F, respectively). This was confirmed by western blot analysis, which revealed a similarly significant increase in the levels of both *ADAR* and *ADAR1b* proteins in the resilient group compared to the susceptible group (*p < 0.05, Fig. 2E, H, G, I, respectively). Pearson’s correlation test revealed that the exploration and the hyperarousal measures significantly correlated with *ADAR* protein expression (*r* = −0.7 and *p = 0.04, *r* = −0.7 and *p = 0.02, respectively) and the social interaction test demonstrated a highly correlative trend with the *ADARb1* protein expression (*r* = −0.6 and *p* = 0.07).

### Alleviation of susceptible behavior by an intra-brain-injection of 5-HT2CR specific antagonist into the CeA

**Fig. 3:**
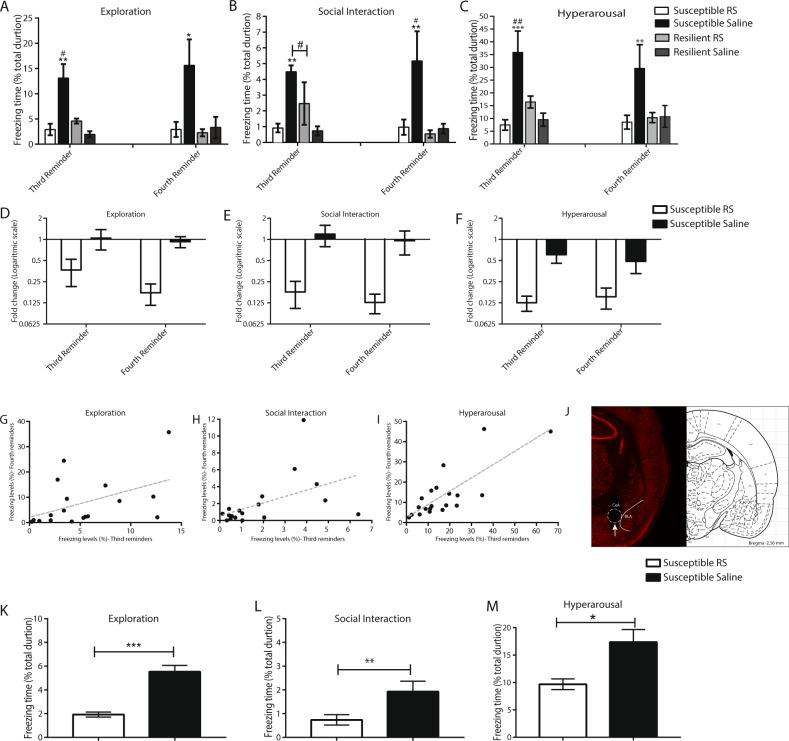
Effect of RS-102221 antagonist treatment on PTSD-like behaviors. Susceptible and resilient animals received one injection of RS-102221 or control vehicle 15 min before the third trauma reminder. Freezing behavior was assessed following the third reminder and fourth reminder in (**A**) exploration, (**B**) social interaction, and (**C**) hyperarousal tests. Bars represent freezing levels after the third and the fourth reminders showing a significant attenuation in freezing behavior of susceptible-RS-treated animals vs. susceptible- control-treated and all of the resilient groups (exploration: third reminder: **p < 0.01 for susceptible-control-treated vs. susceptible-RS-treated and resilient-control-treated animals, *p < 0.05 susceptible-control-treated vs. resilient-RS-treated animals and *p < 0.05 for susceptible-RA-treated vs. resilient-SAM and resilient- RA- treated groups. Fourth reminder: *p < 0.05 for susceptible-RA-treated vs. all other experimental animals; social interaction: third reminder: **p < 0.001 for susceptible-control-treated vs. susceptible-RS and resilient-RS groups and ^#^*p* < 0.05 for susceptible-control-treated vs. resilient-RS treated group. Fourth reminder: **p < 0.01 for susceptible-control-treated vs. all resilient groups and ^#^*p* < 0.05 for susceptible-RA-treated vs. susceptible-control-treated animals; hyperarousal: third reminder: ***p < 0.001 for susceptible-control-treated vs. susceptible-RS-treated and resilient-control-treated groups, ^##^*p* < 0.01 susceptible-control-treated vs. resilient-RS-treated animals. Fourth reminder: **p < 0.01 for susceptible-control-treated vs. all other experimental animals. Data presented as mean ± SEM; n = 4-5 per group. (**D**–**F**) Fold change of freezing behavior in the susceptible-treated-groups in the third and fourth reminders compared to the freezing levels in the second reminder in (**D**) exploration, (**E**) social interaction, and (**F**) hyperarousal behavioral test. (**G**–**I**) Pearson’s product-moment correlation between the third and the fourth reminders in the following tests: (**G**) exploration (**H**) social interaction and (**I**) hyperarousal (r > 0.45;*p < 0.05). (**J**) Left: Image showing PI staining within the CeA. The overlay of the amygdala sub-nuclei demonstrates that the cannula placement is specific to the CeA. The arrow points to the tip of the injection needle. Right: Corresponding section from rat stereotaxic atlas (Paxinos and Watson). (**K**–**M**) Susceptible animals received one systemic injection of RS-102221 or control vehicle 30 min before the third trauma reminder. Freezing behavior was assessed following the third reminder in (**K**) exploration (***p < 0.001) (**L**) social interaction (**p < 0.01) (**M**) hyperarousal (*p < 0.05) tests comparing susceptible group treated with RS-102221 to susceptible group treated with vehicle control. Data presented as mean + SEM; *n* = 4-5 per group.

As the next step, we examined whether the observation that the resilient group had higher levels of the VNV isoform could be translated into a therapeutic strategy. For this purpose, we injected into the CeA (15 min before the third trauma reminder) the RS-102221, a specific antagonist to the 5-HT2CR that was previously tested in a variety of mood disorders such as depression^53,54^. Like editing, the antagonist might be expected to inhibit the activity of the receptor. Interestingly, treatment with RS-102221 significantly attenuated the freezing behavior in susceptible animals in all three behavioral scenarios compared with untreated susceptible controls (*p < 0.05, Fig. 3A–F), (see Supplementary and Methods for full details). Moreover, this effect on PTSD-like behavior in all three scenarios was maintained for three weeks after the single injection-treatment (fourth reminder-day 77) (*p < 0.05, Fig. 3A–F). Pearson’s product-moment correlation test revealed a high correlation between the third and fourth reminders in the different behavioral scenarios (*r* < 0.45; *p < 0.05, Fig. 3G–I). The cannula placement in the CeA is presented in Fig. 3J.

### Alleviation of susceptible behavior by systemic injection of 5-HTR2C specific antagonist

In order to translate our results into a systemic treatment, another group of animals was subjected to the behavioral protocol of the PTSD-like animal model to depict a new group of susceptible animals, as described before. This group was divided into two sub-groups. One received a single injection of RS-102221, and the other received a vehicle as control (1 ml/kg), 30 min before the third trauma reminder^55^. Behavior was measured immediately after the third reminder. The results indicated that RS-102221 treatment significantly attenuated freezing behaviors in susceptible animals compared to treated vehicle controls, immediately after the third reminder in all three behavioral tests (*p < 0.05, Fig. 3K–M), (see Supplementary and Methods for full details).

## Discussion

In the present study, we revealed that animals that were resistant to a traumatic event, compared with susceptible animals, had a significantly higher abundance of the VNV RNA edited isoform of the 5-HT2CR in the CeA. This alteration was associated with higher expression in protein levels of *ADAR* and *ADARb1*. Since no changes in the expression levels of 5-HT2CR transcript were shown, we believe that the behavioral profile of the resilient group, at least partly, stems from altering its editing. To emphasize the role of the observed changes, showing that high editing levels of the VNV isoform lead to resilience, we evaluated the behavioral response after an injection of a specific 5-HT2CR antagonist, RS-102221, directly into the CeA. The results showed that a single injection significantly attenuated the freezing response in all three behavioral situations tested. Interestingly, the relief of the stress response of susceptible animals was maintained for the long-term, when a following reminder of the trauma was tested. This treatment did not alter the behavior of the resilient group, suggesting that the effect was specific for susceptible animals, with no negative effects on animals that did not exhibit PTSD-like behavior.

Moreover, systemic injection of RS-102221 attenuated the susceptible behavior profile in all the three examined tests, suggesting a translational approach to our results.

Importantly, our findings are not in accordance with studies that associate 5-HT2CR agonists with greater fear. Specifically, the effect of Lorcaserin and WAY-163909 on dopaminergic release^16,17^ and other studies showing that transgenic mice having only the fully edited VGV isoform of 5-HT2CR, which thereby overexpress the receptor in the brain, displayed greater fear expression, extensive fear extinction deficits, and fear generalization^56^. It is possible that editing of 5-HT2CR in different brain sites or different cell types might result in different apparent behavior. In our study, we focused on the amygdala. Nonetheless, tight cross-talk is found between this region and other related brain regions, such as the BNST^41^, parahippocampal gyrus, orbitofrontal cortex, sensorimotor cortex, the thalamus, and the anterior cingulate cortex^43^, that may differently respond to trauma. Modulating the 5-HT2CR activity in these regions exerts different downstream signaling pathways such as the brain-derived neurotrophic factor (BDNF)–tyrosine kinase B (TrkB) pathway, the glutamatergic N-methyl-D-aspartate (NMDA) receptor pathway, and the renin-angiotensin system pathway. These signaling pathways may have different effect PTSD-like display^57^.

Alternatively, it might be that the fully edited isoform functions differently than the partially edited one. Intriguingly, these data may suggest that the heritability of an editing setup may express a variety of functional intensities of the 5-HT2CR that are important to cope with stress. Thereby, dominance of edited VGV may be found in people that are more prone to develop PTSD when exposed to a trauma event. This should be verified in future studies.

It is noteworthy that the 5-HT2CR regulated by *ADAR* enzymes is not the only participant in anxiety-related disorders. Simmones and Karanovic have previously identified a role for *ADARb1* and 5-HTR2C in the pathophysiology of major depressive suicide victims, thereby linking genetic and epigenetic factors to an elevated risk of suicide^58,59^.

The PTSD-like animal model used in this study generated a long-lasting susceptibility phenotypic behavior that was accompanied by the downregulation of ADAR enzymes combined with a decrease in the level of the VNV isoform of the 5-HT2CR. Our results demonstrate that blocking the 5-HT2CR by injecting RS-102221 antagonist into the CeA significantly attenuated the PTSD-like behavior of susceptible animals, supporting the hypothesis that the serotonin neurotransmission, via 5-HT2CR, plays a causal role in inducing anxiety-like and susceptibility behavior. Our observations are consistent with previous reports that highlighted the relationship between RNA editing of the 5-HT2CR and neuropsychiatric disorders, specifically with impaired serotonergic tone in PTSD. These studies showed that desensitization of the 5-HT2CR in serotonin transporter (SERT) knockout mice reduced the anxiety phenotype^60^. In this context, an increase in the VNV compared with the INI isoform, in the CeA, can cause a loss of 5-HT2CR activity by reducing the ligand efficacy of the G-coupled protein downstream. For this reason, 5-HT2CR antagonists are often used as pharmacological treatments for generalized anxiety disorders^61,62^ and were previously examined in a rat model for depression^53,54^ (in contrast to agonists, which induce panic attacks in PTSD patients^63^).

Taken together, our results support the suggestion of a causal role for the CeA as a critical brain region for the expression of PTSD-like behaviors and reveal the importance of specific 5-HT2CR -RNA editing as one molecule out of a wide range of targets with the potential to be edited. The specific cell type expressing the RNA editing changes of the 5-HT2CR was not examined. Therefore, a possible explanation for our results may suggest that the RNA editing changes observed in the resilience group may involve glutamatergic neurotransmission. As was previously reported, RS-102221 may target the 5-HT2C receptors present on glutamatergic cells, particularly of the CeA. Moreover, it is plausible that receptors other than 5-HT2CR could be altered by changes in ADARs’ expression and may participate in resilient behavior. These receptors may include glutamate receptors. This was previously reported in the study by Brande-Eilat and colleagues, showing that acquisition of conditioned freezing was associated with changes in expression levels of ADARs followed by RNA editing of glutamate ionotropic receptor kainate type subunit 1 (Grik1)^64^. Further studies examining the specific cell type expressing the 5-HT2CR and other targets of ADARs may provide a deeper understanding of the mechanistic basis of PTSD. The present study explored other targets of ADARs, CACNA1D, and Grik2, but not Grik1.

In Conclusion, the current study introduces a new approach to our understanding of PTSD. We took advantage of a unique PTSD-induced animal model, in comparison with genetic-models^56,65^, that together provide a novel opportunity to converge on the role of RNA editing mechanism in the context of stress-related disorders. Findings from such models demonstrate the value of an unbiased, broad screening analysis of the RNA editing mechanism in different gene networks that may be reprogrammed in psychiatric diseases, by identifying a differential outcome in animals with PTSD-like susceptibility behavior compared to resilience. Our approach was to isolate candidate therapeutic targets by screening a wide variety of sites that could be involved in RNA editing and PTSD and examining the specific behavioral characteristics of each subject. Taken together, our results suggest a causal role for the 5-HT2CR and RNA editing as regulated by the ADAR enzymes in the CeA, for at least partly the expression of PTSD-like behavior. The relief produced by an antagonistic injection for this receptor in both the short and long term and when administered systemically after the traumatic event and its related reminders, suggests that this direction could have future therapeutic potential for PTSD patients.

## Supplementary information

supplementary materials and methods
