# Supplementary material for: RNA editing of the 5-HT2C receptor in the central nucleus of the amygdala is involved in resilience behavior
Source: Transl Psychiatry. 2021 Feb 24;11:137. doi: 10.1038/s41398-021-01252-7 (PMC7904784; doi:10.1038/s41398-021-01252-7)
Supplement: Supplementary file 1 — supplementary materials and methods [file 41398_2021_1252_MOESM1_ESM.pdf]

Supplementary Materials and Methods

Behavioral Procedures

The model used in this research is based on Kesner and Elharrarr et[1,2].The model consists of several stages encompassing 8 weeks (56 days), as described below and presented in Figure 1A.

Habituation

Experimental and naive rats were habituated to their home cage for 7 days, and then habituated (5 minutes per day, for 7 days) to an open field apparatus (90 × 90 × 30 cm Plexiglass box), together with the companion rat from their home cage.

The open field apparatus was viewed by a camera. The video and computer equipment were situated in a separate room in which all video and observation analyses were performed.

Baseline

After 14 days of habituation, baseline freezing levels were measured prior to 'trauma' exposure. Behavioral parameters and measurements are described below (Behavioral Measurements section).

Initial Exposure

One week later (Day 7), experimental rats were exposed to 'trauma', i.e. predator (cat) scent. Each rat was placed for 30 min in a clean plastic cylinder (diameter: 30 cm, height: 33 cm) containing 125 ml of well-soiled cat litter, used by a cat during the 24 h prior to the experiment. After exposure to trauma, the rat was transferred to the open field, and three behavioral parameters were measured (as described in the Behavioral Measurements section below). The cylinder was cleaned between each test. All the litter introduced during the initial exposure tests was soiled by the same cat. Temperature and humidity conditions in the trauma exposure area were identical to those in the housing cages and open field. The

companion rats were not exposed to trauma, and were housed in separate cages in a separate housing area during all experiments.

First Reminder

One week later (day 14), rats were re-exposed for 30 minutes to litter with the same texture, but without the cat scent. Behavioral parameters were subsequently tested (as described below).

Second Reminder

Three weeks later (day 35), rats were again exposed for 30 min to litter with the same texture and without cat scent. Behavioral parameters were subsequently tested (as described below). Behavioral data for this testing stage were analyzed according to the criteria detailed below. Based on the obtained results, experimental rats were categorized as either susceptible or resilient.

Third Reminder

On day 56, rats were again exposed for 30 min to litter with the same texture and without cat scent. Behavioral parameters were subsequently tested (as described below).

Fourth Reminder

On day 98, rats were again exposed for 30 min to litter with the same texture and without cat scent. Behavioral parameters were subsequently tested (as described below).

Behavioral Measurements

After exposure to litter (with or without cat scent), each rat was placed in the open field and assessed for freezing behavior (the amount of time the animal remained completely immobile, for at least 2 s), under three consecutive behavioral conditions: alone (termed "exploration"; 5 min), with their habituated companion ("social interaction"; 5 min), and then during post-startle response after exposure to a loud noise ("hyperarousal"; 5 min). The loud noise was a 36.57 ± 0.3 dB pick/scale. An over baseline noise of 55 ± 0.5 dB

measured by Quest instrument (Quest Technologies, model 2900), calibrated by QC 10 calibrator 114 dB at 1000 Hz, was broadcast to the tested rats during the first 5 min of hyperarousal. The tested rats were not removed from the open field nor touched during the three testing conditions. Behavioral parameters were videotaped and monitored using Observer apparatus and software (Noldus, The Netherlands).

Behavioral Data Analysis: Criteria for Subdivision to Susceptible and Resilient Groups

Baseline behavioral data were analyzed by the Explore procedure in SPSS 11 (IBM Software), in order to define the interquartile range of the population for each behavioral parameter. Results inside the upper range and lower range limits were considered as the resilient (non-PTSD-like) baseline. Deviations from this range were used to retrospectively define extreme behavior (based on results of testing after the second trauma reminder). According to the determined range, animals were categorized as either ‘susceptible’ (exhibiting PTSD-like behavior above the normal baseline in all three conditions), or ‘resilient’ (animals exhibiting at least one behavior in the normal baseline range).

Compiling the target set of A-to-G editing sites

The Fluidigm access array (Fl-AA) system enables the simultaneous amplification of at least 48 different target regions across a 48 sample panel on a single microfluidic device. Thus, 2,304 separate PCR reactions are performed simultaneously, followed by in-parallel next-generation sequencing, allowing the precise quantification of A/G ratios. The basic protocol for the (Fl-AA) system was run in a singleplex PCR mode that limits the number of primer pairs used for the amplification of target regions to 48 per run[3]. We employed a selection process to determine the 48 target regions. We designed the primer set to amplify targets that are evolutionarily conserved editing sites and that reside within the coding region of genes, preferably on editing sites that result in amino acid substitutions[4].

Amplifying targeted editing sites using microfluidic-based multiplex PCR (mmPCR) and next-generation sequencing

To distinguish between the susceptible and resilient samples, primers were designed using Primer3.0 [http://frodo.wi.mit.edu/] and the 454 fusion primer design tool [http://eu.idtdna.com/scitools/applications/fusionprimers/default.aspx (IDT, Coralville, IA)]. Using the mmPCR method, we automatically assembled unique PCR reactions, each reaction including a portion from one of the samples screened with each one of the 48 primer pairs. The mmPCR amplification and tagging strategy is based on two consecutive PCR reactions, each performed with specific fusion PCR primers. The first PCR is performed "on chip" and generates amplicons of interest containing the target sites, which are flanked by designed common sequences [CS1 (fused to the forward primer)/CS2 (fused to the reverse primer)]. The second "off chip" PCR is performed on a thermal cycler and uses the first "on chip" PCR products as templates. The amplicons, now containing the CS regions conjoined (by the previous PCR), can be assigned sample-specific barcodes and Ion-Torrent PGM adaptors, thus making all 48 mini-libraries compatible for in-parallel NGS. Singleplex primer (4 µl, 4 µM per primer in 1X AA-loading buffer) was loaded into the primer inlets of the 21.48 Access Array IFC (Fluidigm, San Francisco, CA, USA). To prepare the cDNA templates, 2.25 µl of each cDNA sample was added to 2.75 µl of the pre-sample mix containing the following enzyme and reagents from the Roche FastStart High Fidelity PCR System: 0.5µl of 10X FastStart High Fidelity Reaction Buffer wo/Mg, 0.5 µl DMSO (5%), 0.1 µl 10mM PCR Grade Nucleotide Mix (200 µM), 0.9 µl 25 mM $MgCl_2$ (4.5 mM), 0.25 µl 20X Access Array Loading Reagent (Fluidigm, San Francisco, CA, USA), 0.05 µl FastStart High Fidelity Enzyme Blend, and 0.7 µl of PCR grade water into the sample inlets of the 21.48 Access Array IFC (Fluidigm, San Francisco, CA, USA). After loading both samples and primers via IFC Controller AX (Fluidigm) script, the IFC was subjected to 40 cycles of thermal cycling using an FC1 Cycler (Fluidigm) with the

following program: 50°C for 2:00 min; 70°C for 20:00 min; and 95°C 10 min. For 10 cycles: 95°C for 15 s; 59.5°C for 30 s; and 72°C for 1 min. For 4 cycles: 95°C for 15 s; 80°C for 30 s; 59.5°C for 30 s; and 72°C for 1 min. For 10 cycles: 95°C for 15 s; 59.5°C for 30 s; and 72°C for 1 min. For 4 cycles: 95°C for 15 s; 80°C for 30 s; 60°C for 30 s; and 72°C for 1 min. For 8 cycles: 95°C for 15 s; 59.5°C for 30 s; and 72°C for 1 min. For 4 cycles: 95°C for 15 s; 80°C for 30 s; 60°C for 30 s; and 72°C for 1 min; ending with 72°C for 3 min. Sample preparation included 1.0 µl of the 1:110-fold diluted PCR products as well as 15 µl of the pre-sample mix containing the following enzyme and reagents from the Roche FastStart High Fidelity PCR System: 2 µl 10X FastStart High Fidelity Reaction Buffer wo/Mg, 1µl DMSO (5%), 0.4 µl 10 mM PCR Grade Nucleotide Mix (200 µM), 3.6 µl 25 mMMgCl2 (4.5 mM), 0.2 µl FastStart High Fidelity Enzyme Blend, and 7.8 µl of PCR grade water. 4 µl of primer mix from the 2 µMAccess Array Barcode Library for Ion Torrent PGM Sequencer– 96 (P/N100-4911), utilizing the B-set; A–BC–CS2, and P1–CS1 barcode primer combination, was added to the sample mix. We used the following PCR program: 95°C for 10 min, 11 cycles of 95°C for 30 s, 60°C for 30 s, 72°C for 1 min, and 72°C for 5 min. All 48-tagged mini-libraries were pooled into a single unified library and purified using the QIAquick PCR purification kit (QIAGEN Sciences, Maryland, USA). The output library was analyzed and quantified in the 2100 Agilent BioAnalyzer system using the HS DNA kit (Agilent Technologies, Santa Clara, CA, USA). After establishing the library dilution factor, the library underwent sequencing preparation using the Ion PGM Template OT2 200 kit, followed by the Ion PGM Sequencing 200-v2 kit, both according to the manufacturers' protocols. The fully processed library was loaded on the Ion 318 chip and sequenced using the Ion-Torrent PGM instructions (Life Technologies, Grand Island, NY 14072, USA).

Bioinformatic sequence analysis

Pre-alignment processing

The sequencing data were downloaded from the machine as a FASTQ files. First, all raw sequences data were de-indexed into 21 samples according to the barcodes used by an in-house script. All reads were trimmed of the universal CS1 and CS2 sequences and all short reads (< 20 nts) were excluded from further analysis. Alignment of the processed reads was made using BWA version 0.7.4-r385, using the MEM option and the parameters: -k 20 -B 3 -O 3 -T 20, for seed in the length of the average primer, and to consider the Ion typical error of small indels.

Alignment process

Alignment was to the Rat RefSeq database (Baylor 3.4/ rn4), where reads that could be aligned to more than one location were omitted from further analysis. We used SAMtools mpileup on the alignment results and ran an in-house script to move the results to the genomic locations from the RefSeqs and then an in-house script to count the number of different nucleotides in each genomic location that had a q-score ≥ 20.

The last stage was to filter the results to a preset set of locations of interest. For each location we evaluated the total number of good quality reads per sample, and the percentage of reads with a “G” at the specified genomic location, was calculated according to the formula; (# of “G” reads / [# of “G” reads + # of “A” reads]).

Analysis of clustered RNA editing sites of the $5HT_{2c}$ receptor

The $5HT_{2c}$ receptor (5HT2CR) known to have 5 sites that are prone to be RNA-edited. These sites are located in close proximity. Therefore, the calculation of the percentage of A/G does not fully reveal the actual editing percentage and may provide null information on all of the possible editing events that may occur. Based on Khermesh et al.[3] we performed isoform cluster analysis of the 5HT2CR generated from the generated

mmPCR-seq data. The RNA editing of each site of the receptor did not exhibit significant changes in the CeA between the susceptible and resilient groups (Figure 2A). Hence, we looked at the abundance of each of the transcript variants of the receptor in order to look for changes in the frequency (%) of the different isoforms. The reads were aligned in order to get the information of each genomic location of the original reads. By a bioinformatic in house program script, we find the editing sites in the 5HT2CR cluster in each read. We used only reads that included all cluster editing sites. For each sample we summed the different combinations of the actual editing locations and calculated the frequency percentage from the total number of reads that covered all the locations for each isoform. Only isoforms that exhibit frequency > 5% were included in the analysis.

Quantitative Real-Time Polymerase Chain Reaction (qRT-PCR) Analysis

ADAR family enzymes; Adar adenosine deaminase, RNA-specific (*ADAR*) and *ADARb1* adenosine deaminase, RNA-specific, B1 *(ADARb1)* and the $5HT_{2c}R$ from the CeA of susceptible and resilient groups, were quantified by reverse transcribing RNA samples to generate cDNA pools (qScript cDNA Synthesis, Quanta BioSciences). These were then used as templates for quantitative real-time PCR analysis. Relative transcript levels were determined by the 7900HT Fast Real-Time PCR System (Applied Biosystems). Triplicates of each cDNA sample were PCR- amplified using the PerfeCTa SYBR Green FastMix (Quanta BioSciences). Reaction protocols were as follows: 30 s at 95°C for enzyme activation followed by 40 cycles of 5 s at 95°C and 30 s at 60°C. Melting curve analysis was used to examine the specificity of the amplification products.

Two set of primers were used for each gene in order to neutralize any possible genomic DNA contamination. In addition, we used two different housekeeping genes (β-Actin and Hprt1) for reference.

Primers for detected genes:

**Gene**	**Forward** Primer	**Reverse** Primer
ADAR (skip)	CAGCTGGAGCTGCACTTGA	AAGAGGATGTTTTCTCGCAGCC
ADAR (same)	CGGGTCTTCAGTACCGGATG	TTAGTGGGCCCCGTGC
ADARb1 (skip)	CCCTGCTCAGGTTTCTATACGC	TTCAGCCGGAACCCACC
ADARb1 (same)	CGGCAGCCAGGAAAGTCT	CCCACAGCTCCTCTTCCC
5HT2CR	ATCGCTGGACCGGTATGTAG	TCACGAACACTTTGCTTTCG
**β-Actin**	AGGCCAACCGTGAAAAGATG	ACCAGAGGCATACAGGGACAA
**Hprt1**	TGTTTGTGTCATCAGCGAAAGTG	ATTCAACTTGCCGCTGTCTTTTA

Western blotting

Whole-cell proteins were extracted from central amygdala (CeA) of susceptible or resilient animals in RIPA buffer including protease inhibitors (Termo Fischer Scientific, MA, USA). Lysates were centrifuged for 8 min at 14,000 rpm at 4°C, and the supernatant was collected. Protein concentration was determined by Bradford analysis (Bio-Rad Protein Assay Dye Reagent Concentrate, Bio-Rad, Hercules, CA, USA). A total of 20 µg protein extract was loaded per lane on 7.5% SDS polyacrylamide gel. After electrophoresis, proteins were transferred to a nitrocellulose membrane (BIO-RAD, Hercules, CA, USA), and the membrane was blocked for 1 h in tris-buffered solution [0.1% Tween (TBST) with 5% skim milk]. Next, the membrane was incubated in TBST with 5% skim milk containing the appropriate primary antibody: anti-ADAR diluted 1:250 ab16138 (Abcam, Cambridge, UK), anti-ADAR1b diluted 1:750 ab64830 (Abcam, Cambridge, UK) or anti-β-Actin dilution 1:5000 ab8227 (Abcam, Cambridge, UK). After washing 3 × 5 min with TBST, the secondary

antibody diluted 1:1000 1:2500 or 1:3000 [rabbit anti-sheep IgG-H&P(HP): ab6747, or goat anti-rabbit IgG-H&L(HRP) preadsorbed: ab7090 (Abcam, Cambridge, UK)] was incubated for 1 h in TBST with 5% skim milk. Membranes were washed 3 × 5 min with TBST and read using the ECL- Signal West Pico Chemiluminescent Substrate according to the manufacturer' s instructions (BIO-RAD, Hercules, CA, USA).

Surgery and cannula implantation

To investigate the effect of RS-102221 treatment on freezing behavior (PTSD-like behavior), rats were anesthetized with ketamine-hydrochloride (100 mg/kg) and xylazine (10-mg/kg; i.p.) one day after the second trauma reminder. A guide cannula (30 gauge) was implanted bilaterally 1 mm above the central amygdala, sealed with a cannula dummy (Plastics One), and secured to the skull with screws and dental acrylic cement. Coordinates of the cannula relative to Bregma[5] were as follows: CeA: anterior –2.56, lateral –4/+4, ventral –7 mm. Rimadyl (2-mg/kg) was injected (s.c.) after surgery. Rats were allowed to recover from surgery for at least 14 days before the third reminder.

Drugs

5HT2CR antagonist, RS-102221 was from Tocris (Bristol, UK). RS-102221 was prepared at to 100mM in DMSO and was then diluted to a final concentration of 5μM in artificial cerebrospinal fluid (aCSF).

Fluorescent staining

In order to validate the cannula placement, rats were anesthetized and then perfused intracardially with phosphate-buffered saline (PBS) followed by 4% paraformaldehyde in PBS. Brains were removed and immersed in 4% paraformaldehyde for 24 hours at 4^0C and then in PBS with 30% sucrose at 40C for 48 hours. Brains were then frozen on dry ice and sliced to collect the CeA (40μ section) with a cryostat microtome at -20^0C

and collected in sodium azide for immunostaining processing. Sections were first rinsed 5 times (10 min each wash) with PBST+0.1%Triton with agitation. Next, sections were stained with propidium iodide (PI) in PBST+0.1%Triton (1:50). Sections were then mounted on glass slides and were coverslip.

Fluorescent image was captured with a LEICA confocal microscope equipped with a digital camera using a × 10 objective.

Statistical Analysis

Characterization of susceptible and resilient rats exposed to trauma in the incubation of fear model

Sprague-Dawley rats (n = 60) were exposed to ‘trauma’ and to three subsequent trauma reminders (Figure 1A). Freezing behavior was then measured in three behavioral scenarios (exploration, social interaction and, hyperarousal). Susceptibility to trauma (PTSD-like behavior) of each rat was compared against the range of the population in the three behavioral tests (Figure 1B-D and Supplementary Methods, Behavioral Procedure and Statistical Analysis sections). The results defined two unambiguous subpopulations, namely, susceptible and resilient. Exploration, social interaction and hyperarousal data were analyzed by two-way ANOVA with Bonferroni's correction. During the exploration test, a two-way ANOVA comparing freezing behavior over the four time points (within subjects) and the two groups (susceptible and resilient) revealed a main effect of group ($F[1,186] = 13.76$; $p = 0.0004$), main effect of time ($F[3,186]=1.96$; $p = 0.0003$) and group x time interaction ($F[1,186] = 13.76$; $p = 0.0004$). Bonferroni's post hoc correction revealed significant differences in the freezing behavior of susceptible versus resilient animals at the time of exposure ($p < 0.05$) and the second reminder ($p < 0.001$) time point (Figure 1B). Analysis of the social interaction test, by two-way ANOVA comparing freezing behavior over the four

time points (within subjects) and the two groups (susceptible and resilient) revealed a main effect of group ($F[1,186] = 18.71$; $p < 0.0001$), main effect of time ($F[3,186] = 16.65$; $p < 0.0001$) and group x time interaction ($F[3,186] = 6.38$; $p = 0.0004$). Bonferroni's post hoc correction revealed significant differences in the freezing behavior of susceptible versus resilient rats after the first and second trauma reminders ($p < 0.001$, Figure 1C). Analysis of the hyperarousal test by a two-way ANOVA comparing the freezing behavior over the four time points (within subjects) and the two groups (susceptible and resilient groups), revealed a main effect of group ($F[1,186] = 17.40$; $p < 0.0001$), main effect of time ($F[3,186] = 46.42$; $p < 0.0001$) and group x time interaction ($F[3,186] = 5.32$; $p = 0.0015$). Bonferroni's post hoc correction showed that susceptible rats had significantly different freezing behavior compared to resilient rats after the first and second trauma reminders ($p < 0.05$ and $p < 0.001$, respectively, Figure 1D). In addition, the distribution data for the second reminder time point revealed an increase in PTSD-like behavior from baseline in the susceptible group, for all three behavioral tests, but not for the resilient rats. Pearson product-moment correlation between exploration with social interaction (Figure 1E), exploration with hyperarousal (Figure 1F) and social interaction with hyperarousal (Figure 1G) were $r = 0.3348$, 0.2826, and 0.2722; $p = 0.0069$, $p = 0.0215$ and $p = 0.0296$ respectively.

Analysis of clustered RNA editing sites of the $5HT_{2c}$ receptor

A two-way ANOVA analysis with the Bonferroni's correction revealed a significant difference in the abundance of the VNV isoform of the 5HT2CR comparing between susceptible and resilient animals. Analysis revealed a main effect of interaction ($F[4,98] = 2.989$; $p = 0.0225$), main effect of isoform ($F[4,98]=26.94$; $p <0.0001$) and no main effect of group ($F[1,98] = 0.1461$; $p = 0.7031$). The Bonferroni's post hoc

correction revealed a significant difference in the relative abundance of the VNV isoform between susceptible and resilient groups after the second reminder of the trauma reminders ($p < 0.0084$, Figure 2B).

Relative mRNA expression levels of the $5HT_{2c}$ receptor

qPCR analysis did not reveal a significant difference in mRNA expression levels of the 5HT2CR in the resilient group compared to the susceptible group (Student's *t*-test (11) = 0.3759, p = 0.7141)(Figure 2C).

Relative levels of expression of *ADAR* and *ADAR1b* mRNA and protein

ADAR and *ADAR1b* are both known to regulate the RNA editing process in the brain and are known to act on the specific site of the 5HT2CR to generate the VNV isoform detected in our analysis. qPCR analysis revealed a significantly higher expression of *ADAR* in the resilient group compared to the susceptible group (Student's *t*-test (12) = 2.93, p = 0.0126)(Figure 2D). Similarly, the levels of *ADAR1b* were also higher in the resilient animals compared to the susceptible group (Student's *t*-test (11) = 2.44, p = 0.0327)(Figure 2F). Like the mRNA, protein levels of *ADAR* and *ADAR1b* were both significantly higher in the resilient animals compared to the susceptible group (Student's *t*-test (4) = 3.316, p = 0.0295 and Student's *t*-test (4) = 3.270, p=0.0303, respectively)(Figure 2E and Figure 2G).

Attenuation of Susceptible Behavior by Injection of RS-102221 into the Central Amygdala

The high frequency of the VNV cluster in the central amygdala of the resilient group, raised the question of the role of the 5HT2CR in the regulation of PTSD-like behavior. We therefore treated our experimental animals with one injection of RS-102221, a

specific 5HT2CR antagonist in order to mimic inhibition of signal transduction after serotonin binds to the receptor. For this purpose, another group of animals were subjected to the behavioral protocol of the *"Incubation of fear"* model and categorized as susceptible or resilient as already described. Each class was then divided into 2 sub-groups which received a single bilateral injection of RS-102221 or aCSF (0.2 µl/side) 15 minutes before the third trauma reminder[6]. Behavior was measured immediately after the third reminder. The results indicated that RS-102221 treatment significantly attenuated freezing behavior in susceptible animals compared to the susceptible-aCSF treated controls immediately after the third reminder in all three behavioral tests. For exploration: one-way ANOVA $F[3,14] = 9.959$; $p = 0.0009$) followed by Newman-Keuls comparison test revealed a significant difference in freezing behavior between susceptible-aCSF treated rats compared to the other three experimental groups ($^{**}p < 0.01$) (Figure 3A). For social interaction: one-way ANOVA $F[3,15] = 7.994$; $p = 0.002$) followed by Newman-Keuls comparison test revealed a significant difference in freezing behavior between susceptible-aCSF treated rats compared to the other three experimental groups ($^{**}p < 0.01$ compared to susceptible-RS and resilient-saline treated groups, $^{\#}p<0.05$ compared to resilient- RS treated groups) (Figure 3B). For hyperarousal: one-way ANOVA ($F[3,15] = 10.81$; $p = 0.0005$) followed by Newman-Keuls comparison test revealed a significant difference in freezing behavior in susceptible-aCSF treated rats compared to the other three experimental groups ($^{\#\#}p < 0.01$ and $^{***}p < 0.001$) (Figure 3C). In order to examine the long-term effects of RS-102221 treatment, the experimental animals were housed in their home cage and were exposed to a fourth trauma reminder, 3 weeks after the third reminder. Behavior was measured immediately after the fourth reminder. The results revealed that susceptible- RS-102221 treated animals continued to exhibit non-PTSD-like behavior

even 3 weeks after the single treatment administered 15 minutes before the third trauma reminder. Freezing behavior was significantly attenuated in susceptible antagonist treated animals compared to the susceptible-aCSF treated animal in all three behavioral tests immediately after the fourth reminder. For exploration: one-way ANOVA (F[3,13] = 5.090; p = 0.0151) followed by the Newman-Keuls comparison test revealed significant difference in freezing behavior between susceptible-aCSF treated rats compared to the other three experimental groups (*p < 0.015) (Figure 3A). For social interaction: one-way ANOVA (F[3,15] = 7.859; p = 0.0022) followed by the Newman-Keuls comparison test revealed a significant difference in freezing behavior between susceptible-aCSF treated rats compared to the other three experimental groups ($^{\#}$p<0.05 and **p < 0.01) (Figure 3B). For hyperarousal: one-way ANOVA (F[3,14] = 3.939; p = 0.0314) followed by the Newman-Keuls comparison test revealed a significant difference in freezing behavior between susceptible-aCSF treated rats compared to the other three experimental groups (**p<0.01) (Figure 3C). . Pearson product-moment correlation between the behavioral tests in the third and fourth reminders revealed high correlation between the behaviors that were measured immediately after the treatment and 3 weeks after the treatment (exploration: r = 0.5945, p = 0.0118; social interaction: r = 0.4842,p = 0.0417 and hyperarousal: r = 0.7920, p < 0.0001) (Figure 3G-I).

Attenuation of Susceptible Behavior by Systemic Injection of RS-102221

In order to translate our results into a systemic treatment, another group of animals were subjected to the behavioral protocol of the *"Incubation of fear"* model and susceptible depicted as described before and divided into two sub-groups. One received a single injection of RS-102221 and the other vehicle as control (1ml/kg) 30 minutes before the third trauma reminder[7] . Behavior was measured immediately after the third reminder. The results indicated that RS-102221 treatment significantly attenuated freezing

behavior in susceptible animals compared to treated controls, immediately after the third reminder in all three behavioral tests. For exploration test Student's *t*-test ((7) = 6.756, p = 0.0003) revealed a significant difference in freezing behavior between susceptible- treated animals compared to susceptible control vehicle treated group. For social interaction test Student's *t*-test ((7) = 5.35, p = 0.0011) revealed a significant difference in freezing behavior between susceptible- treated animals compared to susceptible control vehicle treated group. For hyperarousal test Student's *t*-test ((7) = 3.318, p = 0.0128) revealed a significant difference in freezing behavior between susceptible- treated animals compared to susceptible control vehicle treated group.

References

1 Elharrar E *et al.* Overexpression of corticotropin-releasing factor receptor type 2 in the bed nucleus of stria terminalis improves posttraumatic stress disorder-like symptoms in a model of incubation of fear. *Biol Psychiatry* 2013; **74**: 827–36.

2 Kesner Y *et al.* WFS1 gene as a putative biomarker for development of post-traumatic syndrome in an animal model. *Mol Psychiatry* 2009; **14**: 86–94.

3 Khermesh K *et al.* Reduced levels of protein recoding by A-to-I RNA editing in Alzheimer's disease. *RNA* 2016. doi:10.1261/rna.054627.115.

4 Pinto Y, Cohen HY, Levanon EY. Mammalian conserved ADAR targets comprise only a small fragment of the human editosome. *Genome Biol* 2014; **15**: R5.

5 Paxinos G, Watson C. *The Rat Brain in Stereotaxic Coordinates - The new coronal set*. 2005.

6 McMahon LR, Filip M, Cunningham KA. Differential Regulation of the Mesoaccumbens Circuit by Serotonin 5-Hydroxytryptamine (5-HT) 2A and 5-HT 2C Receptors . *J Neurosci* 2001. doi:10.1523/jneurosci.21-19-07781.2001.

7 Bell R, Duke AA, Gilmore PE, Page D, Bègue L. Anxiolytic-like effects observed in rats exposed to the elevated zero-maze following treatment with 5-HT 2 /5-HT 3 /5-HT 4 ligands. *Sci Rep* 2014. doi:10.1038/srep03881.
